# Behavior of Blood Pressure Variables in Children and Adolescents with Duchenne Muscular Dystrophy

**DOI:** 10.5935/abc.20180085

**Published:** 2018-07

**Authors:** Fabiane R. R. H. Marui, Henrique Tria Bianco, Maria Teresa N. Bombig, Natascha G. F. Palmeira, José M. Thalenberg, Fernando Focaccia Povoa, Maria Cristina de O. Izar, Francisco Antonio H. Fonseca, Acary S. B. de Oliveira, Rui M. S. Povoa

**Affiliations:** Universidade Federal de São Paulo, São Paulo, SP - Brazil

**Keywords:** Cardiovascular Diseases / genetics, Muscular Dystrophy, Duchenne / genetics, Hypertension, Child, Male

## Abstract

**Background:**

Duchenne muscular dystrophy is an X-chromosome-linked genetic disorder (locus Xp21). Involvement of the cardiovascular system is characterized by fibrous degeneration/replacement of myocytes with consequent ventricular hypertrophy and arterial hypertension.

**Objective:**

To assess, by using 24-hour ambulatory blood pressure monitoring, the behavior of blood pressure variables in children and adolescents with a confirmed diagnosis of Duchenne muscular dystrophy.

**Methods:**

Prospective observational cohort study, which selected 46 patients followed up on an outpatient basis, divided according to age groups. Blood pressure was classified according to the age percentile. The monitoring interpretation includes systolic and diastolic blood pressure means, systolic and diastolic blood pressure loads, and nocturnal dipping. The blood pressure means were calculated for the 24-hour, wakefulness and sleep periods. Nocturnal dipping was defined as a drop in blood pressure means during sleep greater than 10%. The significance level adopted was p < 0.05.

**Results:**

Nocturnal dipping for systolic blood pressure was present in 29.9% of the participants. Approximately 53% of them had attenuated nocturnal dipping, and 15%, reverse nocturnal dipping. The age groups of 9-11 years and 6-8 years had the greatest percentage of attenuation, 19.1% and 14.9%, respectively. Regarding diastolic blood pressure, nocturnal dipping was identified in 53.2% of the children, being extreme in 27.7% of those in the age group of 6-11 years.

**Conclusions:**

The early diagnosis of blood pressure changes can allow the appropriate and specific therapy, aimed at increasing the life expectancy of patients with Duchenne muscular dystrophy.

## Introduction

Duchenne muscular dystrophy (DMD) is an X-chromosome-linked genetic disorder that affects approximately 1 in every 3500 live-born boys.^1^

It is clinically characterized by progressive and irreversible muscle weakness consequent to dystrophin deficiency or absence. It is the most frequent neuromuscular disease in human beings, and, although predominating in the male sex, it is occasionally reported in females due to inactivation or abnormalities of the X chromosome. That anomaly is present in the short arm of the X chromosome (*locus Xp21*). Its global prevalence can reach 63 cases per one million individuals. Duchenne muscular dystrophy has a high spontaneous mutation velocity, and approximately one third of the cases are estimated to be due to new mutations.^2-4^ The first clinical signs manifest at an early age as frequent falls, difficulty climbing stairs, running and getting up from a lying or sitting position, and mainly calf hypertrophy. The muscle impairment is symmetric, initiates in the pelvic girdle muscles (hip and legs) and reaches the upper limbs.

In addition, cardiomyopathy is frequent in DMD. While some studies have estimated its incidence in 25% at the age of 6 years, and 59% at the age of 10 years, others have reported its beginning at the age of 14 and 15 years.^5,6^ Cardiac involvement occurs in 90% of the patients, being the cause of death in 50% of them. However, its clinical identification can be hindered by severe muscle weakness and thoracic deformities. Cardiac histological changes include hypertrophy of myocytes and myocardial fibrosis, with replacement with connective and fatty tissues.^7^ Dystrophin deficiency or absence in cardiomyocytes hinders the function of membrane ion channels, notably in sarcolemma, which is activated by stretching, responding to mechanical stress. When cardiomyocytes, with or without dystrophin deficiency, are stretched during ventricular filling, the ion channels do not open properly, increasing calcium influx. Excessive intracellular calcium activates a group of calcium-induced proteases, the calpains, which degrade troponin I and hinder contraction.^8-10^

The importance of the cardiomyopathy in children with DMD has grown in past decades, mainly due to the increase in survival, consequent to advances in ventilatory and orthopedic supports.^11^ In the absence of ventilatory intervention, death usually occurs by the end of the second or beginning of the third decade. Diastolic dysfunction can be present even before systolic dysfunction is detected. The use of drugs that act on the renin-angiotensin-aldosterone axis, such as angiotensin-converting-enzyme inhibitors or angiotensin-receptor blockers, should be considered, aiming at reducing afterload before symptom onset.^12^

Ambulatory Blood Pressure Monitoring (ABPM) allows indirect and intermittent blood pressure (BP) recording for 24 hours, during wakefulness and sleep. In adults, ABPM is a well-established diagnostic and follow-up method, considered "gold standard" in BP assessment.^13^ In 2008, the American Heart Association (AHA) published recommendations for the use of ABPM in the pediatric population, which were reviewed in 2014.^14,15^ Many recommendations of ABPM use for adults can be applied to children. Because of the difficulty of conducting randomized clinical trials in the pediatric population, the recommendations used are based on expert opinions. However, it is worth considering some aspects, such as equipment selection, which should be light (weight between 168 and 457 grams), with appropriate cuff size.^16^

The use of corticosteroid, known to increase BP, has been widely studied. Left and/or right ventricular hypertrophy can cause arterial hypertension (AH) and/or pulmonary hypertension or mitral and/or tricuspid regurgitation, sometimes culminating in ventricular failure.^17,18^ A study by Braat et al. has assessed the renal function of 20 individuals with DMD and undergoing ABPM, 9 of whom had elevated BP (over the 95th percentile), 8 of whom were on corticosteroids, and 13 had no nocturnal dipping (ND), 10 of whom were on corticosteroids.^19^

Knowing BP behavior in such patients is fundamental, mainly because it enables early treatment, contributing to improve the quality of life, aiming at reducing the high morbidity rates of those patients. Thus, our study aimed at assessing the behavior of BP variables, by using 24-hour ABPM, in children and adolescents diagnosed with DMD, followed up at a university-affiliated outpatient clinic specialized in muscular dystrophies.

## Methods

This is a descriptive study comprising all 46 boys with a confirmed diagnosis of DMD followed up on an outpatient basis. Because DMD is a rare disease, we chose to assess all children and adolescents followed up on a university-affiliated outpatient clinic. The boys were divided into five age groups, considering the distribution of normal BP levels for age, according to the previously reported AHA suggestion.^14,15^ The research project was approved by the local Ethics Committee, the information was provided by the parents or guardians, and written informed consent was provided by all participants. Prior to ABPM, the patients' clinical history was collected, and the height and weight measured in the children who could walk, while, for wheelchair-bound patients, historical height was used. Blood pressure was measured at the medical office with the OMROM digital device (HEM 742INT^®^ model) and appropriate cuff size, respecting the proportion width/length 1:2, corresponding to 40% of arm circumference and at least 80% of its length. The Spacelabs 90207^®^ ABPM monitor was installed on the "nondominant" arm, with appropriate cuff size, by a trained nurse, and programmed to take BP every 15 minutes during wakefulness and every 30 minutes during sleep. The parents/guardians received a diary to record the most important events in 24 hours, mainly the bedtime and wake-up time.

The following variables were assessed in ABPM interpretation: systolic and diastolic BP (SBP and DBP, respectively) means; systolic and diastolic blood pressure loads (SBPL and DBPL, respectively); and ND. The SBP and DBP means were calculated for the 24-hour, wakefulness and sleep periods. The SBPL and DBPL were calculated considering the proportion of readings over the 95th percentile. Nocturnal dipping was defined as a drop greater than 10% in BP means during sleep. All parameters were compared to the normal range values to determine whether BP was normal or elevated, as was the presence or absence of ND. In addition, ND was stratified as follows: "present", BP drop during sleep between 10% and 20% as compared to wakefulness; "absent", no BP drop during sleep; "attenuated", BP drop > 0% and < 10% during sleep; "reverse", BP during sleep higher than that during wakefulness; and "extreme", BP drop > 20%.

### Statistical analysis

The continuous variables with normal distribution were presented as means ± standard deviation, while those without normal distribution were presented as medians and interquartile range. The categorical variables were presented as absolute numbers and percentages. The significance level adopted in the statistical analyses was 0.05. Kolmogorov-Smirnov test was used to assess the normality of the variables, and Pearson chi-square test was used to assess the association between corticosteroid use and BP classification.

We used as comparator the BP values of the 95th percentile from the AHA recommendations, as shown in Table 1, which classified children and adolescents according to age groups. Values of *p* <0.05 were considered significant. All statistical analyses were performed with the SPSS software, version 17.0 (SPSS Inc. Chicago, IL, USA)^®^.

**Table 1:** Classification of blood pressure (BP) levels in children

Classification	Office BP	Mean SBP and DBP on ABPM	Blood pressure loads (SBP and DBP)
Normal BP	< 90^th^ percentile	< 95^th^ percentile	< 25%
White coat hypertension	≥ 95^th^ percentile	< 95^th^ percentile	< 25%
Prehypertension	≥ 90^th^ percentile or > 120/80 mm Hg	< 95^th^ percentile	≥ 25%
Masked hypertension	< 95^th^ percentile	> 95^th^ percentile	≥ 25%
Ambulatory hypertension	> 95^th^ percentile	> 95^th^ percentile	25-50%
Severe ambulatory hypertension	> 95^th^ percentile	> 95^th^ percentile	> 50%

Adapted from: A Scientific Statement From the American Heart Association. Hypertension.15 SPB: systolic blood pressure; DBP: diastolic blood pressure; ABPM: ambulatory blood pressure monitoring.

## Results

Table 2 shows the major characteristics of the participants with DMD. Of the 46 children, 57.4% were wheelchair-bound, 69.6% were on corticosteroids, and 6.4% had been previously diagnosed with AH, as reported by their parents/guardians. The diagnosis of DMD was established at around 7 years of age, while the first symptoms appeared approximately at the age of 2.7 years. Other family members were reported to have DMD in 6.4% of the cases. A significant part (63.8%) of the participants was undergoing specific physical therapy, and 40.5% used some type of respiratory support, such as artificial manual breathing unit (AMBU) and/or bilevel positive airway pressure (bipap).

**Table 2:** Baseline characteristics of the patients

Age groups	
3 - 5 years, n (%)	3 (6.5)
6 - 8 years, n (%)	15 (32.6)
9 - 11 years, n (%)	18 (39.1)
12 - 14 years, n (%)	7 (15.2)
15 - 17 years, n (%)	3 (6.5)
**Clinical characteristics**	
Previous diagnosis of SAH,* n (%)	3 (6.5)
Other family members with DMD^†^	3 (6.5)
Use of bipap^‡^	6 (13)
Use of AMBU	12 (26)
Wheelchair-bound	26 (56.5)
Motor physical therapy	29 (63)
Use of corticosteroid	32 (69.6)
Age of the first symptoms of DMD, md (IQR)	7 (5-8)
Age of diagnosis of DMD, md (IQR)	2.5 (1.2-4.5)

SAH*: systemic arterial hypertension; DMD†: Duchenne muscular dystrophy; bipap‡: Bilevel Positive Airway Pressure. Data expressed as numbers (n) and percentages (%); age in median (md) and interquartile range (IQR).

Table 3 shows BP behavior, distribution of the SBP and DBP means with their respective standard deviations in the five age groups, as well as SBPL and DBPL. Regarding SBP, ND was present in 29.9% of the children with DMD. More than half of the participants (53.1%) had attenuated ND, while 15% had reverse ND. The age groups of 9-11 years and 6-8 years concentrated the highest percentage of ND attenuation, 19.1% and 14.9% respectively. Regarding DBP, ND was present in 53.2% of the boys; 27.7% had extreme ND (highest percentage in the age group of 6-11 years: 19.1%), while 14.9% had attenuated ND.

**Table 3:** Distribution of the blood pressure variables (office and ABPM) according to age groups

Age groups (years)	3-5 years n = 3	6-8 years n = 15	9-11 years n = 18	12-14 years n = 7	15-17 years n = 3
Office SBP* (mm Hg)	117.3 ± 17.0	118.3 ± 14.6	118.2 ± 22.2	119.8 ± 18.2	123.3 ± 12.6
Office DBP^†^ (mm Hg)	69.3 ± 9.5	73.2 ± 8.4	76.4 ± 16.6	71.4 ± 9.1	74.3 ± 4.5
ABPM: 24h SBP (mm Hg)	122.6 ± 20.0	117.7 ± 15.6	119.3 ± 19.6	117.4 ± 8.3	114.3 ± 8.5
ABPM: 24h DBP (mm Hg)	73.3 ± 10.1	71.2 ± 15.6	71.2 ± 13.3	71.7 ± 15.1	69.3 ± 5.9
ABPM: wakefulness SBP (mm Hg)	125.0 ± 19.5	121.0 ± 13.7	121.3 ± 19.8	120.1 ± 8.3	117.3 ± 6.5
ABPM: wakefulness DBP (mm Hg)	76.7 ± 13.0	75.5 ± 8.7	71.4 ± 13.6	74.8 ± 8.3	73 ± 4.4
ABPM: sleep SBP (mm Hg)	119 ± 20.1	111.7 ± 18.1	114.2 ± 19.8	110.4 ± 10.8	109 ± 8.9
ABPM: sleep DBP (mm Hg)	66.7 ± 10.7	61.2 ± 18.7	66.2 ± 13.8	62.9 ± 6.7	59.7 ± 8.1
24h SBPL^‡^ (> 50%), n (%)	2 (66.6)	5 (33.3)	7 (38.9)	0 (0)	0 (0)
24h DBPL^§^ (> 50%), n (%)	1 (33.3)	7 (46.7)	7 (38.9)	3 (21.4)	0 (0)
Wakefulness SBPL (> 50%), n (%)	2 (66.6)	5 (33.3)	6 (33.3)	0 (0)	0 (0)
Wakefulness DBPL (> 50%), n (%)	1 (33.3)	5 (33.3)	5 (27.8)	1 (14.3)	0 (0)
Sleep SBPL (> 50%), n (%)	2 (66.6)	7 (46.7)	4 (22.2)	0 (0)	0 (0)
Sleep DBPL (> 50%), n (%)	1 (33.3)	2 (13.3)	3 (16.7)	1 (14.3)	0 (0)

SBP*: systolic blood pressure; DBP†: diastolic blood pressure; SBPL‡: systolic blood pressure load; DBPL§: diastolic blood pressure load. Data presented as numbers (n) and percentages; and mean ± standard deviation.

For BP stratification, the BP measurements taken at the office and on ABPM were considered. Although the recommendations of the specialized guidelines suggest classifying BP in one of the ABPM periods (wakefulness or sleep) or in 24 hours, we classified BP in all periods (Table 4). Regarding the use of corticosteroids, there was no association between corticosteroid use and BP classification in 24 hours (p = 0.904), during wakefulness (p = 0.720) and sleep (p = 0.996).

**Table 4:** Distribution of the participants with Duchenne muscular dystrophy according to blood pressure classification on ABPM during 24hours, wakefulness and sleep

Classification	24h ABPM n (%)		Wakefulness ABPM n (%)		Sleep ABPM n (%)	
Normal	13	28.3	17	36.9	16	34.8
Normal with BPL* > 25%	2	4.3	1	2.2	1	2.2
Prehypertension	6	13.0	4	8.7	7	15.2
Prehypertension without increased BPL	4	8.7	0	0	0	0
White coat SAH^†^	9	19.6	10	21.7	6	13
Masked hypertension	1	2.2	2	4.3	3	6.5
Masked hypertension with SBPL^‡^ > 50%	1	2.2	1	2.2	2	4.3
Severe SAH	11	23.9	10	21.8	8	17.4
High wakefulness or sleep SBP^§^ without increased BPL	0	0	1	2.2	0	0
SAH only on ABPM	0	0	0	0	2	4.3
SAH with SBPL < 25%	0	0	0	0	1	2.2
Total	46	100	46	100	46	100

BPL*: blood pressure load; SAH†: systemic arterial hypertension; SBPL‡: systolic blood pressure load; SBP§: systolic blood pressure. Data presented as numbers (n) and percentages.

## Discussion

Duchenne muscular dystrophy is a disease of poor prognosis, whose survival extends to the second decade of life.^20^ However, the advances in treatment, such as non-invasive ventilation and physical therapy, have enabled boys with DMD to reach 30 years of age. Considering this increase in life expectancy, other aspects, in addition to neuromuscular impairment, need to be assessed.^20^

Although office BP classification in pediatrics was standardized by the National High Blood Pressure Education Program in 2004, the classification of BP obtained from ABPM in children and adolescents has not been standardized. Thus, we used the recommendations based on expert opinions, such as those published by the AHA in 2008. Blood pressure classification in children, according to those recommendations, should consider, in addition to measurements taken at the office, those from ABPM during 24 hours, wakefulness or sleep, and SBPL or DBPL.^21,22^

A study on the BP of boys with DMD has reported the prevalence and correlations of low BP levels measured at the office with a possible autonomic dysfunction due to DMD.^23^ Regarding office BP measurement, more than 50% of the boys had stage 1 or 2 AH, and 12.8% had borderline BP levels. The VII Brazilian Guidelines on Hypertension replaced the term 'borderline' with 'prehypertension', and estimates its prevalence between 10% and 15% of the pediatric population. However, established AH affects 3% to 5% of the children.^24^ In our study, the highest SBP mean during wakefulness was 126.7 ± 10.0 in the age group of 12-14 years, followed by 122 ± 18.6 for office BP in children aged 6-8 years. Considering the percentile for age, the BP mean on ABPM during wakefulness was within the expected range. However, the BP mean at the office was above the 95th percentile. Regarding DBP, the highest mean was observed in the 95^th^ percentile (78.5 ± 12.4) in the age group of 6-8 years on ABPM during wakefulness, followed by 77.3 ± 9.1 in the age group of 15-17 years on ABPM, which is above the estimated percentile. Regarding SBPL and DBPL, all age groups had median > 25% in 24 hours, wakefulness or sleep. Considering only wakefulness, 38.3% of the children had normal BP, 21.3% had severe AH, and 21.3% had white coat hypertension.

The median ND was lower than 10% for SBP and higher than 10% for DBP in all age groups. It is worth noting that 68% of the boys had no 10% ND for SBP. In adults, the absence of ND is considered a risk factor for target-organ damage, in addition to increasing the cardiovascular risk of hypertensive and normotensive individuals. Although the use of corticosteroid can lead to weight gain and BP elevation, it is the only drug that can delay the progression of muscle weakness, reduce the development of scoliosis, and delay respiratory failure. Its mechanism is based on the hypothesis that its anti-inflammatory property and immunosuppressive action promote the proliferation of myoblasts and a reduction in necrosis.^25^ Our study showed no association of corticosteroid use and AH, blood pressure load elevation and ND. Although a significant part of the boys was on prednisone, its administration was intermittent and on the first days of the month.

However, if corticosteroid did not influence BP behavior, what was the factor responsible for the elevated number of hypertensives and BP measurements out of the normal range among those children? The mdx mouse develops X-linked recessive muscular dystrophy (*locus Xp21*) and does not express dystrophin. Although it does not show intense fibrosis and fatty tissue accumulation in muscles, it is considered the most appropriate animal model of DMD. A mechanistic study with those mice has shown that the absence of dystrophin interferes with nitric oxide (NO)-dependent vascular dilatation. When submitted to pressure variations, the vessel showed no adaptation. Because the endothelium is essential for the arteries to adapt to chronic changes in blood flow, in the long run that deficiency might affect the flow-induced vascular remodeling, with consequence to vascular resistence.^26^ Other studies have shown an abnormal sympathetic neurovascular control in dystrophin-deficient muscle, which is evidenced during physical exercise, when sympathetic vasoconstriction is normally absent in active muscles, due to the action of local vasodilating substances, such as NO.^27,28^ The neuronal deficiency of NO sintase, which is reduced in the absence of dystrophin, seems to be the major cause of vasoregulation deficiencies. However, the vascular tone modulation can also be hindered by dystrophin deficiency in arterial smooth muscle cells. Dystrophin is usually expressed in the tunica media of blood vessels, being absent in the vessels of mdx mice.^29,30^

Considering those findings, we might explain the high number of individuals with DMD and BP changes in our study. Another aspect that can be related to endothelial change is the absence or attenuation of ND in those children. Duchenne muscular dystrophy is usually accompanied by changes in the respiratory pattern during sleep, such as obstructive sleep apnea (OSA), causing deleterious effects to the cardiovascular system. In addition, excessive sympathetic activity occurs in OSA, which can contribute to the lack of decrease in BP levels during the night.

### Study limitations

Because DMD is a rare disease, our study can be considered a potential generator of hypotheses. It is worth noting the lack of standardization for ABPM use for children. Thus, we followed the AHA recommendation, which was based on expert opinions. However, such data need to be validated in further studies, with a greater sample power. In addition, it is worth noting the difficulties in measuring the height of the children, because many of them were wheelchair-bound. Thus, we used the historical height, reported by the parents or legal guardians.

Another limitation of our study was the patients' stratification based on age groups, by use of convenience sampling, because of the low incidence of DMD. Because of the difficulty of conducting randomized clinical trials in the pediatric population, the recommendations used were based on expert opinions.

## Conclusion

The analysis of BP variables, obtained mainly from ABPM, is a useful tool to identify patients with DMD at higher risk. Considering the cardiovascular changes of those patients, the early identification of BP changes would allow the appropriate and specific therapeutic intervention. In addition, we suggest the regular and multidisciplinary follow-up of those patients to identify their BP changes, ensuring improvement in their life expectancy and comfort.

## References

[1] Emery AE (1991). Population frequencies of inherited neuromuscular diseases-a
world survey. Neuromuscul Disord.

[2] McDonald CM (2002). Physical activity, health impairments, and disability in
neuromuscular disease. Am J Phys Med Rehabil.

[3] Childers MK, Okamura CS, Bogan DJ, Bogan JR, Sullivan MJ, Kornegay JN (2001). Myofiber. Injury and regeneration in a canine homologue of
Duchenne muscular dystrophy. Am J Phys Med Rehabil.

[4] Kueh SL, Head SI, Morley JW (2008). GABA(A) receptor expression and inhibitory post-synaptic currents
in cerebellar Purkinje cells in dystrophin-deficient mdx
mice. Clin Exp Pharmacol Physiol.

[5] Nigro G, Comi LI, Politano L, Bain RJ (1990). The incidence and evolution of cardiomyopathy in Duchenne
muscular dystrophy. Int J Cardiol.

[6] Connuck DM, Sleeper LA, Colan SD, Cox GF, Towbin JA, Lowe AM, Pediatric Cardiomyopathy Registry Study Group (2008). Characteristics and outcomes of cardiomyopathy in children with
Duchenne or Becker muscular dystrophy: a comparative study from the
Pediatric Cardiomyopathy Registry. Am Heart J.

[7] Cox GF, Kunkel LM (1997). Dystrophies and heart disease. Curr Opin Cardiol.

[8] Feng J, Schaus BJ, Fallavollita JA, Lee TC, Canty Jr JM (2001). Preload induces troponin I degradation independently of
myocardial ischemia. Circulation.

[9] Woolf PJ, Lu S, Cornford-Nairn R, Watson M, Xiao XH, Holroyd SM (2006). Alterations in dihydropyridine receptors in dystrophin-deficient
cardiac muscle. Am J Physiol Heart Circ Physiol.

[10] Williams IA, Allen DG (2007). Intracellular calcium handling in ventricular myocytes from mdx
mice. Am J Physiol Heart Circ Physiol.

[11] American Academy of Pediatrics, Section on Cardiology and Cardiac
Surgery (2005). Cardiovascular health supervision for individuals affected by
Duchenne or Becker muscular dystrophy. Pediatrics.

[12] Viollet L, Thrush PT, Flanigan KM, Mendell JR, Allen HD (2012). Effects of angiotensin-converting enzyme inhibitors and/or beta
blockers on the cardiomyopathy in Duchenne muscular
dystrophy. Am J Cardiol.

[13] Sociedade Brasileira de Cardiologia (2011). V Diretrizes Brasileiras de Monitorização
Ambulatorial da Pressão Arterial (MAPA) e III Diretrizes Brasileiras
de Monitorização Residencial da Pressão Arterial
(MRPA). Rev Bras Hipertens.

[14] Urbina E, Alpert B, Flynn J, Hayman L, Harshfield GA, Jacobson M, American Heart Association Atherosclerosis, Hypertension, and
Obesity in Youth Committee (2008). Ambulatory blood pressure monitoring in children and adolescents:
recommendations for standard assessment: a scientific statement from the
American Heart Association Atherosclerosis, Hypertension, and Obesity in
Youth Committee of the council on cardiovascular disease in the young and
the council for high blood pressure research. Hypertension.

[15] Flynn JT, Daniels SR, Hayman LL, Maahs DM, McCrindle BW, Mitsnefes M, American Heart Association Atherosclerosis, Hypertension and Obesity
in Youth Committee of the Council on Cardiovascular Disease in the
Young (2014). Update: ambulatory blood pressure monitoring in children and
adolescents: a scientific statement from the American Heart Association
Atherosclerosis, Hypertension and Obesity in Youth Committee of the Council
on Cardiovascular Disease in the Young. Hypertension.

[16] Varda NM, Gregoric A (2005). Twenty-four-hour ambulatory blood pressure monitoring in infants
and toddlers. Pediatr Nephrol.

[17] Manzur AY, Kinali M, Muntoni F (2008). Update on the management of Duchenne muscular
dystrophy. Arch Dis Child.

[18] Manzur AY, Kuntzer T, Pike M, Swan AV (2008). Glucocorticoid corticosteroids for Duchenne muscular
dystrophy. Cochrane Database Syst Rev.

[19] Braat E, Hoste L, De Waele L, Gheysens O, Vermeersch P, Goffin K (2015). Renal function in children and adolescents with Duchenne muscular
dystrophy. Neuromuscul Disord.

[20] American Academy of Pediatrics, Section on Cardiology and Cardiac
Surgery (2005). Cardiovascular health supervision for individuals affected by
Duchenne or Becker muscular dystrophy. Pediatrics.

[21] National High Blood Pressure Education Program Working Group on High
Blood Pressure in Children and Adolescents (2004). The fourth report on the diagnosis, evaluation, and treatment of
high blood pressure in children and adolescents. Pediatrics.

[22] O'Brien E, Asmar R, Beilin L, Imai Y, Mancia G, Mengden T (2005). European Society of Hypertension Working Group on Blood Pressure
Monitoring. Practice guidelines of the European Society of Hypertension for
clinic, ambulatory and self blood pressure measurement. J Hypertens.

[23] Masood SA, Kazmouz S, Heydemann P, Li H, Kenny D (2015). Under-recognition of Low Blood Pressure Readings in Patients with
Duchenne Muscular Dystrophy. Pediatr Cardiol.

[24] Malachias MV, Souza WK, Plavnik FL, Rodrigues CI, Brandão AA, Neves MF, Sociedade Brasileira de Cardiologia (2016). 7a Diretriz Brasileira de hipertensão
arterial. Arq Bras Cardiol.

[25] Bushby K, Finkel R, Birnkrant DJ, Case LE, Clemens PR, Cripe L, DMD Care Considerations Working Group (2010). Diagnosis and management of Duchenne muscular dystrophy, part 2:
implementation of multidisciplinary care. Lancet Neurol.

[26] Loufrani L, Levy BI, Henrion D (2002). Defect in microvascular adaptation to chronic changes in blood
flow in mice lacking the gene encoding for dystrophin. Circ Res.

[27] Thomas GD, Sander M, Lau KS, Huang PL, Stull JT, Victor RG (1998). Impaired metabolic modulation of alpha-adrenergic
vasoconstriction in dystrophin-deficient skeletal muscle. Proc Natl Acad Sci USA.

[28] Thomas GD, Shaul PW, Yuhanna IS, Froehner SC, Adams ME (2003). Vasomodulation by skeletal muscle-derived nitric oxide requires
alpha-syntrophin-mediated sarcolemmal localization of neuronal Nitric oxide
synthase. Circ Res.

[29] Brenman JE, Chao DS, Xia H, Aldape K, Bredt DS (1995). Nitric oxide synthase complexed with dystrophin and absent from
skeletal muscle sarcolemma in Duchenne muscular dystrophy. Cell.

[30] Chang WJ, Iannaccone ST, Lau KS, Masters BS, McCabe TJ, McMillan K (1996). Neuronal nitric oxide synthase and dystrophin-deficient muscular
dystrophy. Proc Natl Acad Sci USA.

